# Fluoxetine and Nutrients Removal from Aqueous Solutions by Phycoremediation

**DOI:** 10.3390/ijerph19106081

**Published:** 2022-05-17

**Authors:** Andreia D. M. Silva, Diana F. Fernandes, Sónia A. Figueiredo, Olga M. Freitas, Cristina Delerue-Matos

**Affiliations:** REQUIMTE/LAQV—Associated Laboratory for Green Chemistry (LAQV) of the Network of Chemistry and Technology (REQUIMTE), Instituto Superior de Engenharia do Porto—Politécnico do Porto, Rua Dr. António Bernardino de Almeida 431, 4249-015 Porto, Portugal; andreia.silva@graq.isep.ipp.pt (A.D.M.S.); 1140207@isep.ipp.pt (D.F.F.); cmm@isep.ipp.pt (C.D.-M.)

**Keywords:** *Chlorella vulgaris*, domestic wastewater, immobilization, nutrients, pharmaceuticals, tertiary treatment

## Abstract

The tertiary treatment using microalgae offers an attractive alternative to the removal of low but relevant concentrations of pharmaceuticals from domestic wastewaters. The removal of fluoxetine from aqueous solutions by living and non-living (lyophilized) *Chlorella vulgaris* was assessed. The determination of the pH at the point of zero charge, Fourier transmittance infrared analysis, and scanning electron microscopy were performed to characterize the microalgae biomass. Kinetic and equilibrium experiments were performed. The pseudo-second-order model described the kinetics of fluoxetine. The corresponding kinetic constants indicated that biosorption was faster onto non-living biomass than onto living biomass. The equilibrium results showed that the systems followed the Langmuir isotherm model. The maximum capacity of living microalgae (1.9 ± 0.1 mg·g^−1^) was slightly higher than the non-living microalgae (1.6 ± 0.2 mg·g^−1^). Living *Chlorella vulgaris*, free and immobilized in calcium-alginate, were also used to remove fluoxetine and nutrients (nitrogen and phosphorus) from treated municipal wastewater in a batch system. In both experiments, fluoxetine was completely removed within six days. The total phosphorus (TP) and total nitrogen (TN) removal efficiencies achieved for free and immobilized cells were, null and 65.0 ± 0.1%, and 86.2 ± 0.1% and 81.8 ± 3.1, respectively.

## 1. Introduction

Recent studies regarding the utilization of pharmaceuticals (PhCs) in Europe suggest an increase in antidepressant consumption over time. The most prescribed type of this class is selective serotonin reuptake inhibitors (SSRIs). Fluoxetine (FLX) is a common SSRI and one of the most frequently prescribed antidepressants [1]. After administration, the FLX is subjected to significant hepatic metabolism forming several metabolites. FLX and its metabolites are excreted mainly in the urine and only to a small extent in the faeces [2,3]. Therefore, FLX and its metabolites result in wastewater treatment plants (WWTPs). However, conventional WWTPs were not designed to remove PhCs [4], which makes them a critical pathway for environmental PhCs contamination. Moreover, the release of PhCs into the aquatic environment is not yet subject to regulation, but it is expected that legislation on the discharge of PhCs will be established in the near future.

FLX was designed to produce a specific pharmacological and physiological function, with chemical stability to reach and interact with target molecules [5]. This stability leads to inefficient removal during wastewater treatment. Consequently, FLX has been detected in wastewater effluents and receiving waters at levels ranging from ng·L^−1^ to µg·L^−1^ [1,6]. Despite these low environmental concentrations, the exposition of non-target organisms in the environment has shown that harmful impacts may occur [7,8,9,10,11,12,13,14,15].

Several treatments have been tested to remove PhCs, such as membrane technologies and advanced oxidation processes. However, their cost-effectiveness and ecological impact are considered undesirable [16]. Aware of this problem, the scientific community has been studying alternative tertiary treatments, efficient for PhCs removal, with reasonable costs of operation and maintenance. One alternative that has been gaining increasing interest is phycoremediation [17,18,19,20]. Research on the use of microalgae for the PhCs removal has been growing over the last decade. In addition to the microalgae’s well-known ability to remove organic carbon, nutrients, and heavy metals, they have proven to be a promising eco-friendly, effective, and low-cost alternative for PhCs removal compared with the other treatment processes [17,18].

Coupling PhCs bioremediation with nutrients removal further improves the cost-efficiency of phycoremediation for wastewater treatment since nutrients represent approximately 10% of the costs of microalgae cultivation [21]. In Europe, WWTPs have stringent limits regarding nitrogen and phosphorus removal. They have to meet the Nitrates Directive (91/676/EEC) and the Urban Waste Water Treatment Directive (UWTD) (91/271/EEC). Despite all efforts to date, the control of nitrogen and phosphorus pollution remains one of the most critical environmental issues. It is well known that excess nutrients in water bodies can result in the eutrophication of lakes and rivers.

Although microalgae have promising abilities for wastewater treatment, their use is limited by their difficult separation from the treated effluent. Harvesting methods include physical, chemical or biological methods. However, these methods are too slow or too expensive for cost-effective algal harvesting [22]. As an alternative, immobilization techniques have been explored. Microalgae immobilization in alginate, a natural polysaccharide matrix, is one the most widely employed due to its non-toxicity, permeability, low cost, and high affinity for water. Furthermore, microalgae cells do not suffer extreme physical-chemical conditions changes during the immobilization process [23].

In this context, this study aimed to evaluate the potential of living and non-living (lyophilized) *Chlorella vulgaris* biomass to remove FLX from aqueous solutions under batch operation. The ability of free and immobilized living *Chlorella vulgaris* to simultaneously remove FLX and nutrients from treated municipal wastewater in a batch system was also assessed. This unicellular green microalga was chosen due to its rapid growth rate and tolerance to the severe environmental conditions found in municipal effluents [24,25]. Previous studies showed that this microalga has the potential to remove nutrients [26] and PhCs from wastewater [27]. Moreover, the biomass produced during wastewater treatment may also be used to produce carbon-neutral products, such as biofuels, bioplastics, and exopolysaccharides. Integration of wastewater treatment with microalgal bio-refinery will not only achieve the wastewater treatment purpose, but also generate revenue and support a circular and sustainable bio-economy [18].

## 2. Materials and Methods

### 2.1. Materials

#### 2.1.1. Microalgae and Culture Conditions

*Chlorella vulgaris*, Ref. 211-8L from SAG, cultures were maintained in glass flasks of flat-bottom containing the standard medium Organisation for Economic Co-operation and Development Test Guidelines (OECD TG 201) [28], at room temperature, light/dark photoperiod (24:0 h), a light intensity of 2685 ± 215 lux provided by fluorescent lamps (90 lm·W^−1^) (OSRAM L36 W/865, Munich, Germany) and measured with a digital light meter (Lutron, Lx-1102, Taiwan, China). The culture was continuously aerated by filtered air (Acro^®^50, 0.2 μm PTFE membrane) using an aquarium sparger (Tetratec^®^, APS 50, Melle, Germany).

To obtain non-living biomass, the *Chlorella vulgaris* culture was centrifuged at 8000 rpm (Heraeus Megafuge 16R Centrifuge, Thermo Fisher Scientific, Waltham, MA, USA) at 4 °C for 10 min. The harvested biomass was frozen at −80 °C (*Haier Bio-Medical Ultra*-*Low Temperature Freezer*, DW-86L578J, Qingdao, China) for 24 h, freeze-dried (Edwards) at −44 °C and 0.7 mbar for three days, and stored in a silica gel desiccator. All manipulations were performed under aseptic conditions.

#### 2.1.2. Reagents and Solvents

The pharmaceutical FLX hydrochloride ((RS)-*N*-methyl-3-phenyl-3-[4-(trifluoromethyl)phenoxy]propan-1-amine) (purity > 98%) was obtained from Sigma-Aldrich (Taufkirchen, Germany), its physicochemical properties are listed in Table S1 [29,30] (Supplementary Materials). Stock standard solution of FLX hydrochloride (1300 mg·L^−1^) was prepared on a basis weight in methanol and stored at −20 °C in a dark glass vial.

The information concerning the other reagents is provided in Table S2 (Supplementary Materials). All of the working solutions were prepared with ultrapure water (resistivity of 18.2 MΩ·cm at 25 °C) using a Simplicity 185 system (Millipore, Molsheim, France).

### 2.2. Methods

#### 2.2.1. *Chlorella vulgaris* Biomass Characterization

The pH at the point of zero charge (pH_PZC_), Fourier transmittance infrared (FT-IR) spectra, and scanning electron microscopy with energy dispersive spectroscopy (SEM/EDS) micrographs were used to characterize living and non-living biomass before and after FLX uptake. For the characterization, samples were ground and dried in an oven (Selecta P, 2000208, Barcelona, Spain) at 70.0 °C for 24 h and cooled in a silica gel desiccator.

The pH_PZC_ and FT-IR (Thermo Scientific, Nicolet 6700 FT-IR, MCT/A detector, Waltham, MA, USA) analyses were performed as described by Silva et al. [20]. The SEM/EDS (FEI Quanta 400 FEG ESEM/EDAX Genesis X4M) analysis was performed as described by Monteiro et al. [31].

#### 2.2.2. Immobilization of *Chlorella vulgaris* Cells in Alginate Beads

The immobilization of *Chlorella vulgaris* was performed as described by Lau et al. [32]. Approximately 2022 spherical beads were produced with a diameter of 3.93 ± 0.43 mm (Mitutoyo, Japan). All manipulations were performed under aseptic conditions.

#### 2.2.3. *Chlorella vulgaris* Concentration Measurement

The *Chlorella vulgaris* concentration was monitored by determining the chlorophyll in vivo fluorometrically, as recommended by the United States Environmental Protection Agency (US EPA) [33]. A linear relationship between dry weight, *DW* (mg·mL^−1^), and fluorescence intensity of chlorophyll, *FI*_485/645_ (a.u.), was established for low (Equation (S1)) and high (Equation (S2)) concentrations (Supplementary Materials).

The dry weight was determined by filtering a precise volume of microalgae suspension on previously washed, dried, and pre-weighed (Metter Toledo^®^, NewClassic MF, MS 205DU, Greifensee, Switzerland) glass microfiber filter (Whatman, Grade 934-AH, Maidstone, UK) with a pore size of 1.5 μm and 47 mm diameter. Then, the filter was dried in an oven at 80 °C for 24 h and cooled in a silica gel desiccator until constant weight. Microalgae biomass dry weights were then determined by the difference between the two weights. Dry weights determinations were performed in duplicate. The fluorescence intensity was determined using a microplate reader (BioTek, Synergy HT, Winooski, VT, USA) at the excitation/emission wavelength pair of 485/645 nm. Data acquisition was performed using Gen5 software (version 2.00.18) (BioTek Instruments, Winooski, VT, USA). Fluorescence intensity readings were performed in sextuplicate.

Additionally, the concentration of *Chlorella vulgaris* culture was monitored as cell density for the free and immobilized microalgae, *NC* (cells mL^−1^) and *IC* (cells beads^−1^), respectively, by cell counting with a Neubauer chamber using an optical microscope Leica DM500 (Leica Microsystems, Wetzlar, Germany). For immobilized *Chlorella vulgaris* cells, beads were previously solubilized in a 5% (*w*/*v*) tri-sodium citrate solution at 100 rpm.

#### 2.2.4. Determination of FLX Concentration

The quantification of FLX was performed by HPLC-FLD using a Shimadzu LC system (Shimadzu Corporation, Kyoto, Japan) equipped with a LC-20AD pump, a DGU-20A 5R degasser, a CTO-10AS VP column oven, a SIL-20A HT automatic injector, and a RF-20A-XS fluorescence detector. The chromatographic separation was achieved using a Luna C_18_ column (150 × 4.6 mm, 5 μm particle size) (Phenomenex, Torrance, CA, USA), using the method described by Silva et al. [20]. The identification of the analyte was based on its retention time compared with a standard solution, and the quantification was performed using the external calibration curve method. A linear relationship between peak area and the FLX concentration was established in the range of 1–2000 μg·L^−1^, according to Equation (S3) (Supplementary Materials). The limit of detection (LOD) and limit of quantification (LOQ) were determined on the basis of the signal-to-noise ratio using the analytical response of 3 and 10 times the background noise, respectively. The determined LOD and LOQ for FLX were 10.8 and 35.9 μg·L^−1^, respectively.

#### 2.2.5. Determination of Nutrients (Total Phosphorus and Total Nitrogen) Concentration

The total phosphorus (*TP*) was measured by molecular absorption spectrophotometry (Standard Methods 4500-P, Ascorbic acid method) using an UV-VIS spectrophotometer (HACH DR/2000, Loveland, CO, USA). The total nitrogen (*TN*) was measured by combustion using a TN analyzer (Shimadzu, VCSN, Japan). Samples were previously centrifuged at 8000 rpm for 10 min to separate *Chlorella vulgaris* biomass. Samples and blanks were analyzed in triplicate.

#### 2.2.6. Biosorption Studies

Batch experiments were performed to study the biosorption of FLX by living and non-living *Chlorella vulgaris* biomass. The experiments were performed at room temperature and pH range of 7–8 without further adjustment, considering the typical pH range for domestic wastewater (between 6.5 and 8.0) [34], the pH range favorable to the growth of *Chlorella vulgaris* (between 7.5 and 8.0) [35] and the expected range after the secondary treatment (between 6.5 and 7.5) [36]. The biosorption capacity and the removal efficiency were calculated according to Equations (S4) and (S5), respectively (Supplementary Materials).

##### Kinetic Experiments

The kinetic experiments were performed to reveal the biosorption equilibrium time (*t_e_*) and the adsorption rate for living and non-living biomass. The assays were performed for 120 min, in 250 mL Erlenmeyer flasks with an initial FLX concentration of 560 µg·L^−1^. For living biomass, the biomass concentration was previously determined in a culture sample of the microalga, as described in Section 2.2.3, and a 45 mL of *Chlorella vulgaris* culture was centrifuged at 8000 rpm for 10 min. The harvested suspension of biomass was added to the flasks and stirred at 500 rpm (SBS^®^ Instruments S.A., ACS-160 magnetic stirrer hotplate, Barcelona, Spain). An initial sample was taken before adding the microalgae biomass. Further samples were collected in defined time intervals, centrifuged at 9000 rpm (Thermo Scientific, Heraeus Fresco 21 Microcentrifuge, Dreieich, Germany) for 10 min and the supernatants were collected and analyzed by HPLC-FLD, as described in Section 2.2.4. The pH value was also measured and recorded at all the determined time intervals. The same procedure was followed for non-living biomass, but in this case, an equal amount of biomass was simply weighed before its addition to the FLX solution. Blank experiments (without biomass) were run in parallel with kinetic experiments.

##### Equilibrium Experiments

The equilibrium experiments were performed to determine the biosorption isotherms for living and non-living biomass. The assays were performed in 250 mL Erlenmeyer flasks using the same initial FLX concentration, 560 µg·L^−1^, and the initial biomass concentration was varied. For living biomass, the volumes of *C. vulgaris* culture were previously determined to obtain different biomass weights: 0.5, 1, 2, 7, 15, 30, 50, 70, 100, and 120 mg. The volumes of culture were centrifuged at 8000 rpm at 4 °C for 10 min. The harvested suspension of biomass was then added to the flasks and stirred at 500 rpm for the *t_e_* determined from kinetic experiments. For FLX quantification, samples were taken at the beginning and end of the experiments. Samples were centrifuged at 9000 rpm for 10 min, and supernatants were collected and analyzed by HPLC–FLD, as described in Section 2.2.4. The same procedure was followed for non-living biomass, but in this case, equal amounts of biomass were only weighed before their addition to the FLX solutions. Blank experiments (without biomass) were run in parallel with equilibrium experiments.

##### Data and Statistical Analysis

Three reaction models, the Elovich [37], pseudo-first-order [38], and pseudo-second-order [39] models (see Equations (S6)–(S8), respectively, Supplementary Materials), were fitted to the experimental kinetic results. Five equilibrium models, the Freundlich [40], Langmuir [41], Langmuir–Freundlich (Sips) [42], Redlich–Peterson [43], and Tóth [44] models (see Equations (S9)–(S13), respectively, Supplementary Materials), were fitted to the experimental results.

Non-linear regressions were used in model adjustments using OriginPro software (version 8.5 SR1) (OriginLab Corporation, Northampton, MA, USA). The best fit, suitability, and agreement of non-linear regressions were validated using six statistic parameters: Sum of squares error (SSE), standard error of estimate ($S_{y.x}$), adjusted R-square correlation coefficient ($R_{adj}^{2}$), reduced chi-squared ($\chi_{red}^{2}$), Akaike information criterion (*AIC*), and Bayesian information criterion (*BIC*) tests. 

#### 2.2.7. Removal of FLX and Nutrients from Real Treated Municipal Wastewater

Two batch experiments were performed to evaluate the simultaneous removal of FLX and nutrients (nitrogen and phosphorus) from treated municipal wastewater by free and immobilized living *Chlorella vulgaris*. The characteristics of the treated wastewaters used in the assays are shown in Table S4 (Supplementary Materials).

The assays were performed in closed 500 mL Erlenmeyer flasks with a working volume of 250 mL. FLX was added to treated municipal wastewater to an initial concentration of 750 µg·L^−1^ (>20 times higher than the LOQ), previously inoculated with free or immobilized *Chlorella vulgaris* (in the exponential growth phase). FLX solution was prepared in the OCDE culture medium to guarantee the same concentration of nutrients in all assays. The assays were run at room temperature, under continuous illumination, with a light intensity of 2685 ± 215 lux provided by fluorescent lamps (90 lm·W^−1^), without pH adjustment, and were agitated at 100 rpm on an orbital shaking plate (Bunsen, AO-400, Spain) for nine days. 

Five independent control assays were included and remained under the same conditions (1st, 2nd, 3rd, 4th, and 5th control assays). The purposes of each assay are depicted in Table 1. The composition of free and immobilized *Chlorella vulgaris* assays is shown in Table 2. 

In the free *Chlorella vulgaris* experiments, samples were taken on days 0, 1, 2, 5, 6, 7, 8, and 9 to monitor FLX concentration and chlorophyll fluorescence intensity (*FI*_485/645_) in the main experiment. In all the other assays, samples were taken at the beginning (day 0) and the end (day 9) to monitor temperature, pH, concentrations of *FLX*, *TN*, *TP*, and the *NC*.

In the immobilized *Chlorella vulgaris* experiments, samples were taken at the beginning (day 0) and the end (day 9) to monitor temperature, pH, concentrations of *FLX*, *TN*, *TP*, and the *NC* for all assays. 

All of the experiments were performed in duplicate. All of the manipulations were performed under aseptic conditions. The removal efficiencies and the growth inhibition rate were calculated according to Equations (S5) and (S14), respectively (Supplementary Materials).

#### 2.2.8. Statistical Analysis

Statistical analysis was performed with MedCalc^®^ statistical software (version 12.5.0.0) (Medcalc Software, Ostend, Belgium) and OriginPro software (version 8.5 SR1). The normal distribution of results was tested using the Kolmogorov–Smirnov test, and the outliers’ presence was tested using the generalized extreme studentized deviate (GESD). Removal efficiencies and concentrations of FLX and nutrients were described as mean ± standard error. Differences between means were analyzed using one-way ANOVA followed by Tukey’s test (OriginPro). The statistical significance was defined as a *p*-value < 0.05. Mean ± standard error are shown in the figures (*n* = 2).

## 3. Results and Discussion

### 3.1. Biomass Characterization

#### 3.1.1. FT-IR Results

**Figure 1:**
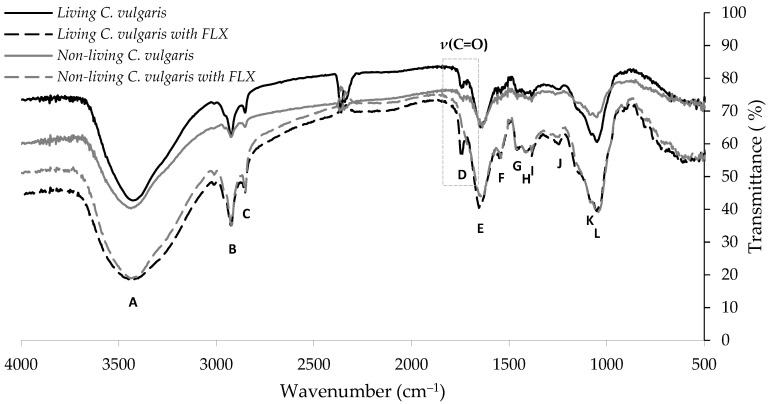
Fourier transform infrared (FT-IR) spectra of living and non-living *Chlorella vulgaris* biomass before and after FLX uptake. The letters A–L indicate the main bands identified in the spectra.

The functional groups present on the *Chlorella vulgaris* biomass surface that may act as binding sites in the uptake process of FLX were identified by FT-IR. FT-IR spectra from living and non-living microalgae biomass before and after FLX uptake are shown in Figure 1. Twelve main bands can be identified and the respective wavenumber assignments are shown in Table S5 (Supplementary Materials). In the region between 4000 and 3100 cm^−1^, single broadband at about 3438 cm^−1^ (band A) is observed, which is assigned to O–H stretching vibrations of water or hydroxyl radicals of polysaccharides, and N–H stretching vibrations of proteins (amide A) [45,46]. The region between 3100 and 2800 cm^−1^ exhibits two bands at about 2923 and 2855 cm^−1^ (bands B and C, respectively) that are assigned to C–H stretching vibrations, more specifically to CH_2_ asymmetric and symmetric stretching vibrations of lipids and carbohydrates [45,46]. Complementary information can be obtained from the region between 1470 and 1350 cm^−1^ where the various deformation modes of these functional groups are found in the spectrum. The region between 1800 and 1500 cm^−1^ is dominated by the conformation-sensitive amide bands. The absorption band at about 1647 cm^−1^ (band E) corresponds to the C=O stretching vibration of amide I, which is associated with proteins [45,46]. The band at about 1565 cm^−1^ (band F) is due to N–H deformation of amide II and C–N deformation of proteins [45,46]. Useful information can also be obtained from the band near 1739 cm^−1^ (band D) essentially resulting from C=O stretching vibrations of the ester functional groups in fatty acids and cellulose [45,47]. Complex absorption profiles are observed between 1500 and 1300 cm^−1^ arising predominantly from CH_2_ and CH_3_ bending modes of lipids, proteins, and ring vibrations of nucleic acids. Bands G and H at about 1462 and 1417 cm^−1^, respectively, are associated with CH_2_ and CH_3_ asymmetric deformations of lipids and proteins, and C–O symmetric stretching vibrations of carboxylic groups. The branching in the band at about 1400 cm^−1^ indicates that two or three methyl groups may be connected to the same carbon atom. Below 1300 cm^−1^, bands at around 1387 and 1250 cm^−1^ (bands I and J, respectively) are assigned to P=O asymmetric stretching vibrations of phosphodiester backbone from nucleic acids and phospholipids [45,47]. The spectral region between 1200 and 900 cm^−1^, bands at about 1080 and 1049 cm^−1^ (bands K and L, respectively) are assigned to P=O asymmetric stretching vibrations of phosphodiester backbone from nucleic acids and to a strongly coupled C–O, C–C stretching and C–O–H, C–O–C deformation vibrations of carbohydrates [45,46].

The FT-IR spectra of living and non-living *Chlorella vulgaris* biomass do not show significant differences between them. This similarity between the spectra allows for inferring that freeze-drying did not introduce changes in the chemical structure of raw *Chlorella vulgaris* biomass as the literature proposes. According to Chen et al. [48], freeze-drying allows the preservation of the cell constituents without varying the biochemical composition significantly.

The FT-IR spectra show small punctual shifts between absorption bands of living and non-living spectra of *Chlorella vulgaris* biomass with and without biosorbed FLX. This may be related to the relatively low proportion of biosorbed FLX comparison to the mass of *Chlorella vulgaris* and/or to the overlapping of other stronger bands in the same wavenumbers. The only exception is the disappearance of the peak at about 1739 cm^−1^ assigned to C=O stretching vibrations (see Figure 1) in the non-living *Chlorella vulgaris* biomass with biosorbed FLX. No other bands allow the identification of the presence of the biosorbed FLX. The bands at about 1329 cm^−1^ characteristic of C–F stretching vibrations from the trifluoromethyl group and the band at about 697 cm^−1^ characteristic of phenyl ring vibrations mono-substituted of FLX are not present [49].

#### 3.1.2. SEM/EDS Results

**Figure 2:**
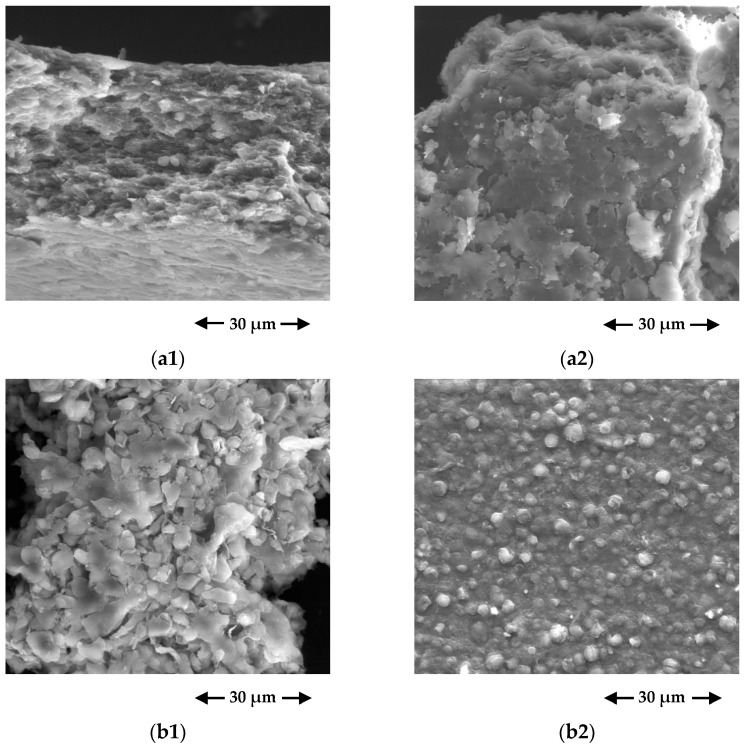
Scanning electron microscopy (SEM) micrographs of living *Chlorella vulgaris* biomass (**a1**) before and (**a2**) after FLX uptake, and non-living *Chlorella vulgaris* biomass (**b1**) before and (**b2**) after FLX uptake (secondary electrons; ×3000; 15 kV; working distance =10.7 mm).

Scanning electron microscopy (SEM) was performed to identify the topological changes in living and non-living *Chlorella vulgaris* cells before and after FLX uptake. SEM micrographs are shown in Figure 2. For both living and non-living cells, it is possible to observe that there were no significant changes after the biosorption assays. Due to the filling of intercellular spaces by the biosorbed FLX, the surface morphology of *Chlorella vulgaris* biomass became more compact.

Energy dispersive spectroscopy (EDS) analysis was performed to identify elemental composition changes in living and non-living *Chlorella vulgaris* cells before and after FLX uptake. The EDS graphs and the respective element analysis are shown in Figure S1 (Supplementary Materials) and Table 3, respectively. The spectra were obtained from several points to ensure the representativeness of the EDS analysis. The working distance of EDX analysis was 10.7 mm, part of this distance corresponds to the Au/Pd film, thus FLX biosorbed molecules at a distance greater than 10.7 mm were not detected. The EDS results reveal the presence of different chemical elements both for living and non-living microalgae cells. The peaks of gold (Au) and palladium (Pd) are associated with the coating film. Before FLX uptake, carbon (C) and oxygen (O) are the predominant chemical elements, while other minerals (sodium (Na), magnesium (Mg), silica (Si), phosphorus (P), sulphur (S), potassium (K), and calcium (Ca)) were detected at trace levels (less than 2%). These elements are characteristic of the composition of organic compounds, such as the *Chlorella vulgaris* biomass. After FLX uptake, no significant changes were observed in the elemental composition.

#### 3.1.3. pH_PZC_ and pH Effect Results

**Figure 3:**
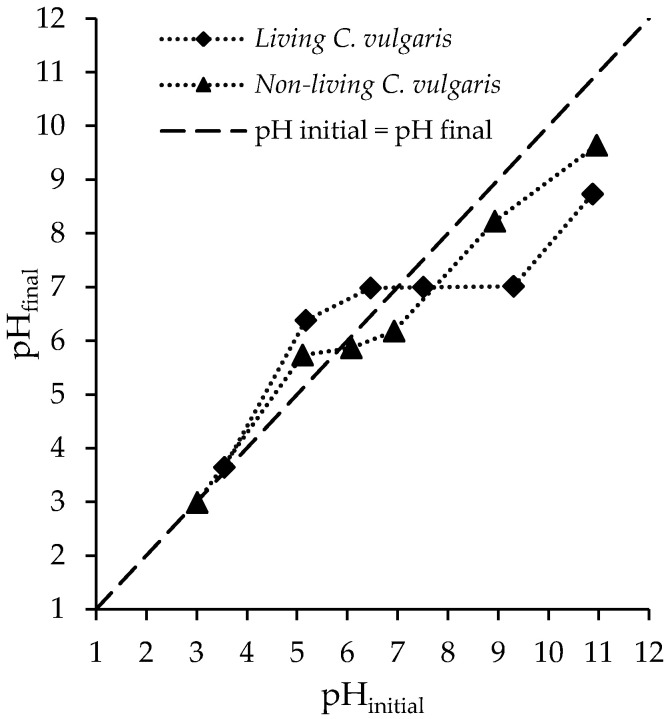
The pH at the point of zero charge (pH_PZC_) determination in living and non-living *Chlorella vulgaris* biomass.

In general, the sorption process in the liquid phase is highly pH-dependant. It determines the *Chlorella vulgaris* biomass surface charge and the dissociation or protonation of the FLX molecules. The pH_PZC_ values for living and non-living *Chlorella vulgaris* biomass were 7.0 and 5.8, respectively (see Figure 3). This difference may be a consequence of the freeze-drying process, which can cause cell lysis, exposing intracellular organelles membranes [48].

At a solution pH lower than pH_PZC_, the net surface charge of *Chlorella vulgaris* biomass is positive, whereas at a higher solution, the pH is negative [50]. FLX (hydrochloride) has an ionizable amino group with a pKa of 9.8 (see Table S1, Supplementary Materials) [29,30]. As can be seen in Figure S2 (Supplementary Materials) [51], there are three ranges, in which boundaries can be defined by pH= pKa - 2 and pH= pKa+2 [52]. For pH values below 7.8, FLX molecules are predominantly positively charged, whereas for pH values above 11.8, FLX molecules are predominantly neutral (see Table S1, Supplementary Materials) [51]. In the pH range between 7.8 and 11.8, the solution is characterized by the coexistence of neutral and positive species. Therefore, attraction forces can be predicted to occur mainly in the pH ranges of 7.0–9.8 and 5.8–9.8 for living and non-living *Chlorella vulgaris* biomass, respectively, where maximum biosorption was expected to occur. The electrostatic or Coulomb forces are the main forces responsible for interactions between sorbents and sorbates. Other types of physical interactions, such as dipole–dipole forces and London–van der Waals, may also act in the biosorption process. However, the strength of these physical interactions is weaker than the electrostatic forces [53].

The effect of solution pH on the FLX biosorption capacity of living and non-living biomass was studied in the pH range 6–9, which was established for wastewater discharge by the Directive 2006/44/EC. It was observed that although the experiments started with different pH values, the final values tended toward the pH_PZC_ values, and therefore the effect of solution pH on the FLX biosorption capacity was not significant. Therefore, the kinetic and equilibrium studies were carried out at pH 7.4 ± 0.3 and 7.6 ± 0.1 for living and non-living biomass, respectively. 

### 3.2. Kinetic and Equilibrium Studies

#### 3.2.1. Kinetic Results

**Figure 4:**
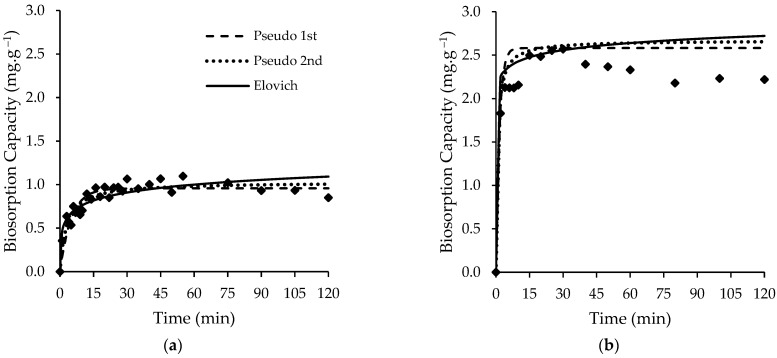
Kinetic experimental data with fitted models for uptake of FLX for (**a**) living *Chlorella vulgaris* biomass and (**b**) non-living *Chlorella vulgaris* biomass.

Fittings of the reaction models (pseudo-first-order, pseudo-second-order, and Elovich equations) to the experimental results are shown together with the experimental results in Figure 4. The kinetic parameters derived from these fittings are depicted in Table 4. Based on the highest $R_{adj}^{2}$ value and the lowest *SSE*, $S_{y.x}$, $\chi_{red}^{2}$, *AIC*, and *BIC* values, it is possible to state that the pseudo-second-order model provides the best fit over the whole time range for both living and non-living *Chlorella vulgaris* biomass. 

As seen in Figure 4, the time interval used (120 min) was largely sufficient to attain the equilibrium, which was about 20 and 15 min for living and non-living cells, respectively, which are inversely proportional to the values of the kinetic constants. The values of the kinetic parameters are depicted in Table 4. In both systems, the high rate of FLX uptake in the initial stage is possibly due to the numerous functional groups’ existence (stated in FT-IR analysis) available for binding. After the initial stage, the FLX uptake rate decreased, which can be attributed to the saturation of binding sites. These results suggest that freeze-drying may have introduced physical changes in *Chlorella vulgaris* biomass. Previous studies [48,54] showed that freeze-drying can increase cell walls’ porosity and average pore diameter. These same studies showed that freeze-drying can also lead to cell lysis exposing intracellular organelles membranes.

#### 3.2.2. Equilibrium Results

**Figure 5:**
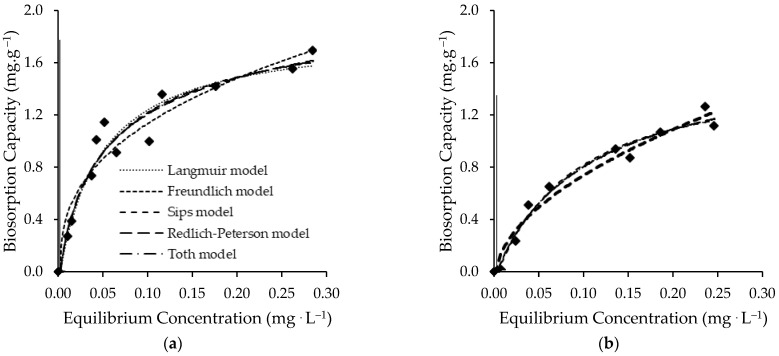
Equilibrium experimental data with fitted models for uptake of FLX for (**a**) living *Chlorella vulgaris* biomass and (**b**) non-living *Chlorella vulgaris* biomass.

Fittings of the Freundlich, Langmuir, Langmuir−Freundlich (Sips), Redlich−Peterson and Tóth models to the experimental results are shown with the experimental results in Figure 5. The equilibrium parameters derived from these fittings are depicted in Table 5. As it can be seen in Figure 5, all of the isotherms have an *L*-type configuration according to Giles classification [55], which shows a monoprotic approach to a limiting value that corresponds theoretically to the complete filling of a surface monolayer.

Although the lowest *SSE* value was obtained for the Redlich−Peterson model, it is considered that the Langmuir model provides the best fit for living and non-living *Chlorella vulgaris* biomass based on the highest $R_{adj}^{2}$ value and the lowest $S_{y.x}$, $\chi_{red}^{2}$, *AIC*, and *BIC* values. Langmuir model is a theoretically derived isotherm based on assumptions that: FLX molecules are adsorbed at a fixed number of well-defined localized sites; each site can hold one FLX molecule; all of the sites are energetically equivalent; and there is no interaction between molecules on neighboring sites [41]. Although the Langmuir model has been stated as the best fit, this is not necessarily proof that the underlying assumptions are all fulfilled. 

The maximum monolayer capacity ($q_{mL}$) of living *Chlorella vulgaris* biomass (1.9 ± 0.1 mg·g^−1^) is slightly higher than the maximum monolayer capacity of non-living *Chlorella vulgaris* biomass (1.6 ± 0.2 mg·g^−1^). The $K_{L}$ values suggest a greater affinity of FLX for living *Chlorella vulgaris* biomass surface, although the values are the same order of magnitude. The observed difference may have resulted from different types of interactions between FLX-biomass.

The blank experiments run in parallel with the other experiments allowed for the verification that about 9% of FLX concentration was abiotically removed, suggesting potential interactions of the PhC with the medium. The remaining removal is expected to be related to sorption mechanisms. Volatilization is not expected to play a significant role in the FLX removal considering its low Henry constant value (≈2.46 × 10^−5^ Pa·m^3^·mol^−1^) and the experimental conditions (stirring, temperature, and contact time) [56].

Similar results were reported in a previous study [57], when living and non-living biomass of *Chlorella vulgaris* were used to remove flutamide, an anticancer PhC, from wastewater. The living biomass showed better performance, considering the biomass amount, pH, and adsorption time. Moreover, in another study [58], living biomass of the microalga *Phaeodactylum tricornutum* was shown to be significantly more effective and efficient than non-living biomass in oxytetracycline removal. 

*Chlorella vulgaris* cell wall consists of a polymer membrane with components as water, microfibril A, cross-linked by hydrogen bonds, and a matrix consisting of proteins and complex polysaccharides [59]. As exposed in FT-IR analysis, these constituents of the cell wall and the other parts of the microalgal cell possess functional groups that have a significant potential for PhCs binding. The biosorption of PhCs onto microalgal biomass comprises two main processes: Hydrophobic interactions between PhCs and microalgal biomass, extracellular polymeric substances or the lipophilic cell membranes of microalgae; and electrostatic interactions between positively charged groups of PhCs and the mainly negatively charged surfaces of microalgae. FLX has a hydrophobic character (log Kow 4.17), and the pH of the assays were 7.4 ± 0.3 and 7.6 ± 0.1 for living and non-living biomass, respectively, which is within the ranges where maximum biosorption was expected to occur (see Section 3.1.3). Therefore, the occurrence of biosorption was favored for both living and non-living *Chlorella vulgaris* biomass. In the case of the living *Chlorella vulgaris* biomass, results suggest that other removal mechanisms, such as bioaccumulation and biodegradation, may have been involved. Indeed, previous publications [17,60] have reported that PhCs can be transported across the microalgal cell membranes into the cells via three major pathways: Passive diffusion, passive-facilitated diffusion, and energy-dependent/active transport. Once inside the microalgal cells, the PhCs may be intracellularly biodegraded. Biosorption is usually the first step of bioaccumulation, but not all PhCs biosorbed on microalgal surfaces can bioaccumulate in cells. PhCs biodegradation may also take place extracellularly. In both cases, biodegradation involves the breakdown of PhCs through microalgae metabolic pathways with possible by-products formation or complete mineralization to carbon dioxide and water. 

The maximum monolayer adsorption capacities obtained for FLX were compared with other sorbents reported in the literature, determined under identical experimental conditions, as can be seen in Table 6 [19,61,62,63]. Compared with the other materials, particularly paper mill sludge and carbon activated, the maximum monolayer capacities obtained in this study are quite low. The maximum monolayer capacities of living and non-living *Chlorella vulgaris* biomass are only comparable to hollow trees and vine biochars.

Despite the low capacities obtained, it would be compatible with the concentrations found in treated domestic wastewaters (between not detected and 72.0 ng·L^−1^ [64]), and moreover the use of the living microalga may bring economic and environmental benefits, namely their low operation costs, continuous growth, and ability to remove nutrients.

### 3.3. Removal of FLX and Nutrients from Real Treated Municipal Wastewaters

#### 3.3.1. Free *Chlorella vulgaris* Assays Results

The potential of free *Chlorella vulgaris* to simultaneously remove FLX and nutrients, phosphorous and nitrogen, from treated municipal wastewater in batch assays was evaluated (consult composition in Table S4, Supplementary Materials). The abiotic parameters, temperature and pH, were monitored during the assay (nine days). The temperature underwent slight variations during the experiment (between 22.0 and 23.0 °C), remaining within the temperature range of 15–25 °C, accepted as optimal for microalgae growth [65]. The results of pH monitorization are shown in Figure S3 (Supplementary Materials). At the beginning of the experiment (day 0), the pH value was 7.18 ± 0.02 for all assays. At the end of the experiment (day 9), pH remained stable in the uninoculated assays (2nd, 3rd, and 5th control assays) and increased in the inoculated assays (main experiment, 1st, and 4th control assays), possibly due to the photosynthetic assimilation of carbon dioxide (CO_2_/O_2_ dynamics), and/or the excretion of alkaline metabolites from the biodegradation of organic matter. 

**Figure 6:**
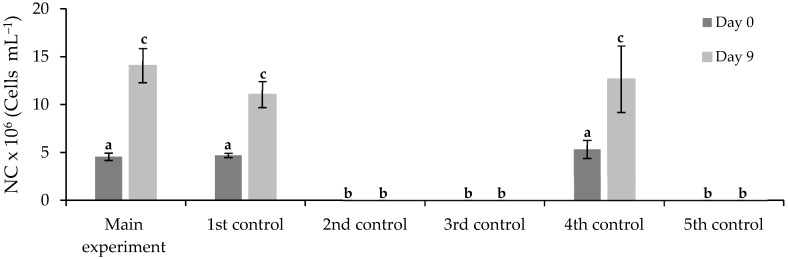
Cell density of free *Chlorella vulgaris* at the beginning (day 0) and the end (day 9) of main and control assays. Different letters indicate significant differences among assays.

The results of cell density (NC) are shown in Figure 6. Differences between means were analyzed using one-way ANOVA followed by Tukey’s test (OriginPro) at the 0.05 significance level. Mean ± standard error are shown, n = 2. There was no microalga in the 2nd, 3rd, and 5th control assays. In the main experiment, the free *Chlorella vulgaris* grew from 4.6 × 10^6^ ± 0.4 ± 10^6^ to 14.1 × 10^7^ ± 0.2 ± 10^7^ cells·mL^−1^ after nine days of biological treatment. The results suggest that the wastewater composition stimulated the *Chlorella vulgaris* growth in the main experiment (when compared with the 4th control without any inhibitors), and the FLX presence did not cause inhibition (in comparison with the 1st control), although the concentration of FLX greatly exceeded the environmentally relevant concentrations reported in the literature [1,6]. This may be explained by the fact that FLX and the organic matter present in the wastewater may have been used as additional organic carbon sources, and due to the supplementary presence of nutrients. It is well known that *Chlorella vulgaris* can grow in autotrophic, mixotrophic, and heterotrophic modes [66].

**Figure 7:**
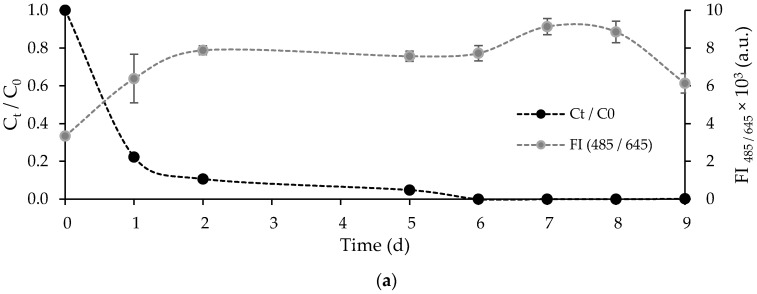

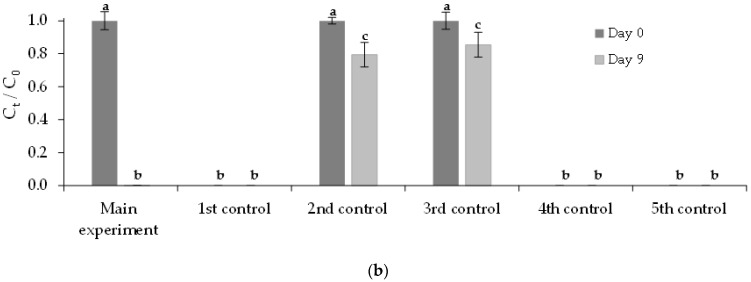
(**a**) Progression curves of normalized FLX concentration and cell density of free *Chlorella vulgaris* over time for the main experiment; and (**b**) normalized FLX concentration at the beginning (day 0) and the end (day 9) of the main experiment and control assays. Different letters indicate significant differences among assays.

The evolution of the FLX concentration, normalized by the initial concentration, $(C_{t}/C_{0}$) and fluorescence intensity of chlorophyll (*FI*_485/645_) over time for the main experiment is shown in Figure 7a. At the beginning of the experiment (day 0), the concentration of FLX was 726 ± 38 µg·L^−1^, and the fluorescence intensity of chlorophyll was 3342 ± 162 a.u. The growth of *Chlorella vulgaris* did not show any lag phase, suggesting a good adaptation to the treated municipal wastewater. The fluorescence intensity increased in the first two days, coinciding with the period of higher average FLX removal rate. The FLX removal rate on the first day was 247 ± 15 mg·L^−1^·d^−1^, decreasing on the second day to 37 ± 7 mg·L^−1^·d^−1^. The fluorescence intensity stabilized between the second and the sixth day, and the mean FLX removal rate decreased to 8.5 ± 1.1 mg·L^−1^·d^−1^. After achieving the complete removal of FLX, the fluorescence intensity increased, reaching its peak value on the seventh day, declining until the end of the experiment (stabilizing in 6135 ± 518 a.u.), possibly, due to nutrient depletion, toxic accumulation, and pH increase (to 9.75 ± 0.01).

In the control assays, the normalized FLX concentration was measured at the beginning (day 0) and the end of the experiment (day 9). The results are shown in Figure 7b. As expected, the normalized FLX concentration was below the LOD in the 1st, 4th, and 5th control assays (no FLX was added). The 2nd and 3rd control assays suggest that the removal of FLX cannot be attributed exclusively to *Chlorella vulgaris* assimilation. The removal efficiencies achieved in the 2nd and 3rd control assays, 20.5 ± 7.4% and 14.3 ± 7.5%, respectively, (*p*-value < 0.05) suggest that indigenous microorganisms from the treated municipal wastewater contributed to the FLX removal (in the 2nd control) and FLX underwent abiotic degradation (in the 3rd control).

At the beginning of the experiment, the *TP* concentration in the main experiment was 1.58 ± 0.02 mg·L^−1^. After nine days of biological treatment, no *TP* removal was achieved. This value respects the discharge limits established by the Urban Waste Water Treatment Directive (UWTD) (91/271/EEC) for *TP* concentration 2 mg·L^−1^ for 10,000–100,000 population equivalent (p.e.), although for more than 100,000 p.e. the limit would be 1 mg·L^−1^.

**Figure 8:**
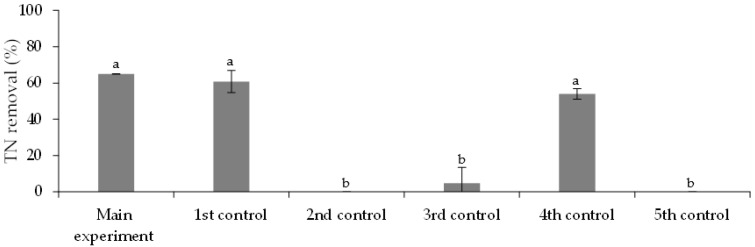
TN removal efficiencies at the end (day 9) of the main experiment and control assays. Different letters indicate significant differences among assays.

The *TN* removal efficiencies are shown in Figure 8. At the beginning of the experiment, the *TN* concentration in the main experiment was 18.3 ± 0.1 mg·L^−1^. After nine days of biological treatment, the removal efficiency achieved was 65.0 ± 0.1%. The removal efficiency achieved in the 1st control assay, 61 ± 6%, is not statistically different (*p*-value > 0.05), suggesting that the FLX presence did not inhibit the nitrogen assimilation by *Chlorella vulgaris*. Conversely, the removal efficiencies achieved in the 2nd and 5th control assays, null values (*p*-value < 0.05), suggest that the nitrogen removal was exclusively due to *Chlorella vulgaris* assimilation, without the indigenous microorganism’s intervention from the treated municipal wastewater (2nd control assay) or abiotic nitrogen removal (5th control assay). The *TN* achieved, 6.4 ± 0.1 mg·L^−1^, is below the discharge limits established by the UWTD, 10 mg·L^−1^ (for 10,000–100,000 p.e.) and 15 mg·L^−1^ (for more than 100,000 p.e.).

In this study, the initial C:N:P ratio (9.6:11.6:1.0) is different from the so-called Redfield ratio (106:16:1) [67]. This may have affected the nutrient removal by *Chlorella vulgaris*. The C:N ratio, in particular, plays an important role in nutrient assimilation. Carbon is the most vital nutrient that is required by microalgae. Probably the characteristics of the wastewater used in the bioremediation experiment were not suitable to meet the requirements of *Chlorella vulgaris* biomass. To accommodate this mismatch and to satisfy the *Chlorella vulgaris* growth condition, a strategy that can be adopted in real WWTP is to supply carbon by sparging carbon dioxide into the culture medium. Additionally, the co-addition of nutrient-rich wastewater may be beneficial. Regarding the N:P ratio, the results suggest that nitrogen removal was not affected, given the final concentration of TN achieved, 6.4 ± 0.1 mg·L^−1^.

#### 3.3.2. Immobilized *Chlorella vulgaris* Assays Results

In this experiment, wastewater with different characteristics was used, with higher levels of organic matter and nutrients (see Table S4, Supplementary Materials). Similar to free *Chlorella vulgaris* experiments, the temperature underwent slight variations during the experiments (between 22.0 and 24.0 °C) and remained within the temperature range of 15–25 °C, accepted as optimal for microalgae growth [65]. The results of pH monitorization are shown in Figure S4 (Supplementary Materials). At the beginning of the experiment (day 0), the mean pH value was 7.81 ± 0.07 for all assays. At the end of the experiment (day 9), the pH increased in the main experiment, 1st, 3rd, and 4th control assays, and decreased in the 2nd and 5th control assays.

**Figure 9:**
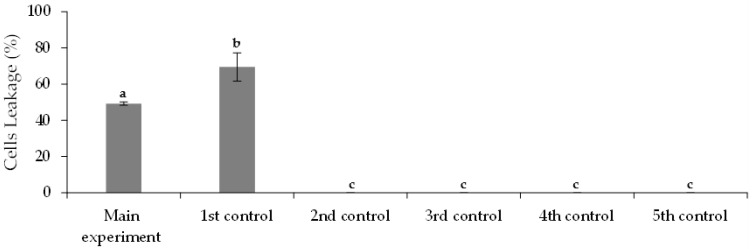
Cells leakage at the end (day 9) of main and control assays. Different letters indicate significant differences among assays.

The integrity of calcium-alginate beads was maintained for five days of the bioremediation experiment. On the sixth day, in the main experiment and 1st control assay (both containing treated municipal wastewater), the beads began to show disruption signs, and the culture medium turned green in color, indicating that the release of microalgae cells from the beads had occurred. The initial mean diameter of beads, 3.93 ± 0.43 mm, was visibly reduced at the end of the bioremediation experiment. In contrast, for the 4th control assay (containing OCDE culture medium), the integrity and diameter of beads were maintained over the experimental time. These results suggest that the beads’ integrity was vulnerable. The results of cell leakage are shown in Figure 9. The cell leakage for the main experiment and 1st control assay are statistically different (*p*-value < 0.05), respectively, 49.2 ± 1.0% and 69.5 ± 7.8%.

**Figure 10:**
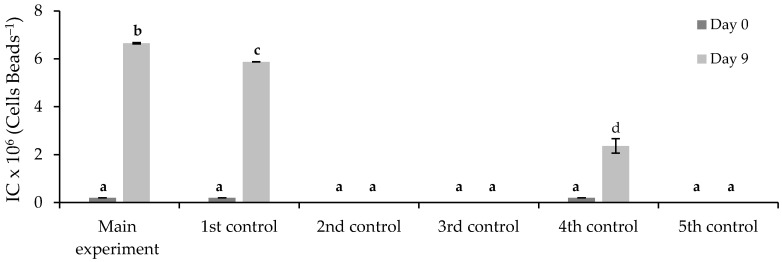
Cell density beads of *Chlorella vulgaris* at the beginning (day 0) and the end (day 9) of main experiment and control assays. Different letters indicate significant differences among assays.

The results of cell density beads (IC) are shown in Figure 10. In the main experiment, the immobilized *Chlorella vulgaris* grew from 1.9 × 10^5^ ± 2.7 × 10^5^ to 6.6 × 10^6^ ± 3.0 × 10^4^ cells·beads^−1^ after nine days of biological treatment. The results suggest that the wastewater composition stimulated the *Chlorella vulgaris* growth, and the FLX presence or the immobilization process did not cause inhibition. 

**Figure 11:**
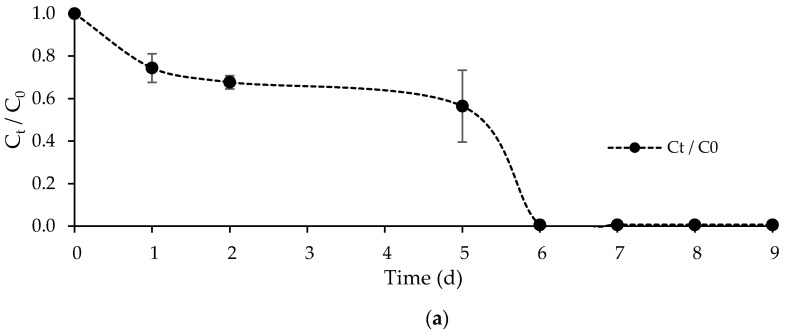

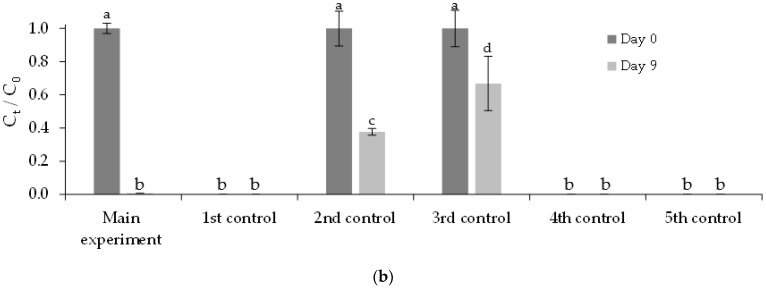
(**a**) Progression curve of normalized FLX concentration over time for the main experiment; and (**b**) normalized FLX concentration at the beginning (day 0) and the end (day 9) of the main experiment and control assays. Different letters indicate significant differences among assays.

The evolution of the FLX concentration, normalized by its initial value, $(C_{t}/C_{0}$) over time for the main experiment is shown in Figure 11a. In the first five days of treatment, the FLX removal rate was 7.7 ± 3.3 mg·L^−1^·d^−1^. On the sixth day of treatment, the FLX concentration decreased abruptly (with a FLX removal rate of 9.9 ± 2.6 mg·L^−1^·d^−1^), achieving the complete PhC removal, probably driven by the beginning of the cell leakage. These results suggest that the immobilization matrix may have limited FLX removal in the first five days of biological treatment. These results also suggest that, for a successful treatment, the immobilized *Chlorella vulgaris* would require a longer retention time and thus, maintaining the integrity of the calcium-alginate beads is a key factor. This drawback may be overcome using strontium salts rather than calcium chloride or by treatment with chitosan to increase beads resistance [23,68]. Alternatively, the concentrations of alginate and Ca^2+^ may also be changed [69]. However, it should be noted that the beads’ thickness increase may reduce the mass transfer of FLX and target nutrients, nitrogen and phosphorus [70].

The normalized FLX concentration at the beginning (day 0) and the end of the experiment (day 9) from all of the assays are shown in Figure 11b. As expected (no FLX was added), the normalized FLX concentration was below the LOD in the 1st, 4th, and 5th control assays. The 2nd and 3rd control assays suggest that the removal of FLX cannot be attributed exclusively to *Chlorella vulgaris* assimilation. The removal efficiencies achieved in the 2nd and 3rd control assays, 62 ± 2% and 32 ± 16%, respectively, (*p*-value < 0.05) suggest that indigenous microorganisms from the treated municipal wastewater contributed to the FLX removal (verified in the 2nd control) and FLX underwent abiotic degradation (in the 3rd control). 

**Figure 12:**
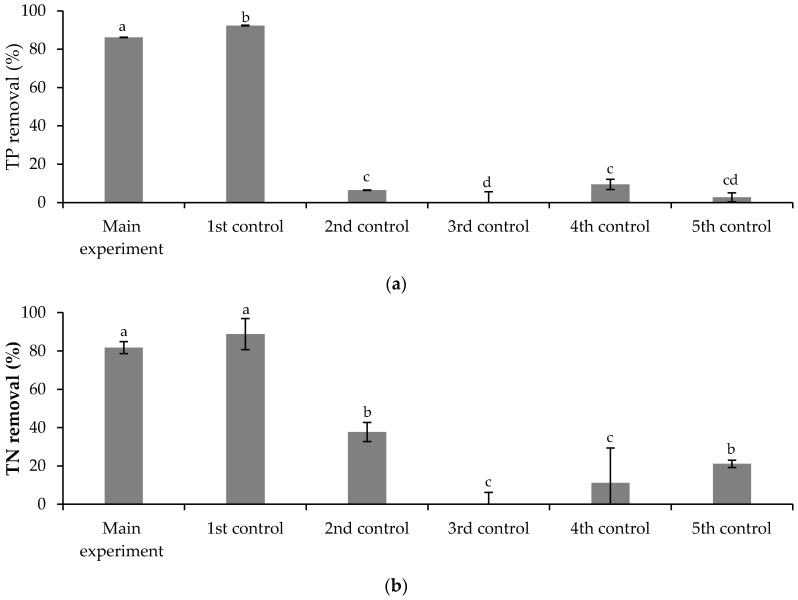
(**a**) TP and (**b**) TN removal efficiencies at the end (day 9) of the main experiment and control assays. Different letters indicate significant differences among assays.

The *TP* removal efficiencies are shown in Figure 12a. At the beginning of the experiment, the *TP* concentration in the main experiment was 21.0 ± 0.7 mg·L^−1^. After nine days of biological treatment, the removal efficiency achieved was 86.2 ± 0.1%. The removal efficiency achieved in the 1st control assay, 92.4 ± 0.2%, (*p*-value < 0.05) suggests that the FLX presence may have inhibited slightly the assimilation of phosphorus by *Chlorella vulgaris*. The removal efficiencies achieved in the 2nd and 5th control assays, 6.4 ± 0.1% and 2.8 ± 2.3%, respectively, (*p*-value < 0.05) suggest that the phosphorus removal was mainly due to *Chlorella vulgaris* assimilation, with a small contribution from the indigenous microorganisms present in the treated municipal wastewater. Although the removal efficiency achieved is above the minimum percentage of reduction, 80% imposed by legislation, the final *TP* concentration, 2.763 ± 0.004 mg·L^−1^, is above the discharge limits established by the UWTD, 2 mg·L^−1^ (10,000–100,000 p.e.) and 1 mg·L^−1^ (more than 100,000 p.e.). 

Regarding *TN*, the removal efficiencies are shown in Figure 12b. At the beginning of the experiment, the *TN* concentration in the main experiment was 49 ± 2 mg·L^−1^. After nine days of biological treatment, the removal efficiency achieved was 82 ± 3%. The removal efficiency achieved in the 1st control assay, 89 ± 8%, is not statistically different (*p*-value > 0.05), suggesting that the FLX presence did not inhibit the nitrogen assimilation by *Chlorella vulgaris*. The removal efficiencies achieved in the 2nd and 5th control assays, 37 ± 5% and 21 ± 2%, respectively, (*p*-value < 0.05) suggest that indigenous microorganisms from the treated municipal wastewater contributed to the nitrogen removal. The final *TN* concentration, 8.6 ± 1.8 mg·L^−1^, is below the discharge limits established by the UWTD, 10 mg·L^−1^ (10,000–100,000 p.e.) and 15 mg·L^−1^ (more than 100,000 p.e.).

In this study, the initial C:N:P ratio (1.4:2.4:1.0) was also different from the Redfield ratio [67]. As previously mentioned, both the C:N and N:P ratios play important roles in nutrient assimilation. The carbon deficit may also have limited nitrogen and phosphorus assimilation by *Chlorella vulgaris* in this experiment. Nevertheless, high nutrient removal efficiencies were achieved. The phosphorus-rich culture medium may have provided higher phosphorus uptake than the metabolic needs of *Chlorella vulgaris* (by “luxury uptake”) [71]. The experimental conditions (e.g., pH 8 or higher) may also have favored abiotic phosphorus removal by precipitation in the main experiment and 1st control assay.

## 4. Conclusions

This study showed that both living and non-living *Chlorella vulgaris* are able to remove the low concentrations of FLX found in treated domestic wastewaters. Moreover, in the experimental conditions applied, the living *Chlorella vulgaris* microalga (free or immobilized) can combine, in one step, the removal of FLX and nutrients from treated domestic wastewater. Despite the several advantages, the immobilization of microalgae in calcium-alginate beads has challenges. For successful wastewater treatment, the immobilization matrix may require a longer retention time, which may increase treatment costs.

In a circular economy approach, the biomass produced during the treatment may be used as a feedstock for third-generation biofuels production and synthesized bioactive molecules can be extracted, fractionated, and valued in an integrated perspective, using a biorefinery concept.

Therefore, the use of *Chlorella vulgar* is biomass is a promising low-cost and eco-friendly alternative to remove FLX and nutrients from aqueous matrices. However, it is important to note that, in this work, the research was focused on the removal of FLX and nutrients conducted in batch systems under controlled laboratory conditions. Further research is required to evaluate the removal of FLX and nutrients in WWTPs. Moreover, attention should be paid to the by-products formation and their potential toxicity.

## Figures and Tables

**Table 1:** Purposes of the assays were carried out for the simultaneous removal of FLX and nutrients from the treated municipal wastewater by free and immobilized *Chlorella vulgaris*.

Assay	Purposes
Main experiment	▪ To assess the bio removal of FLX and nutrients by the *Chlorella vulgaris* and other microorganisms present in the treated wastewater.
1st control	▪ To assess the removal of nutrients by the *Chlorella vulgaris* and other microorganisms present in the treated wastewater in the absence of FLX. ▪ To serve as a control to assess the inhibition of *Chlorella vulgaris* growth by the wastewater components.
2nd control	▪ To assess the bio removal of FLX and nutrients by microorganisms present in the treated wastewater.
3rd control	▪ To serve as a blank, assessing the stability of FLX during the experiment and its potential abiotic degradation.
4th control	▪ To assess the *Chlorella vulgaris* growth in the absence of any potential inhibitor. ▪ To serve as a reference for growth inhibition rate calculations.
5th control	▪ To evaluate any change in the composition of the treated wastewater, possibly due to photo- or biodegradation.

**Table 2:** Composition of assays carried out for the simultaneous removal of FLX and nutrients (nitrogen and phosphorus) from the treated municipal wastewater by free and immobilized *Chlorella vulgaris*.

Assay	Municipal Wastewater (% *v*/*v*)	*Chlorella vulgaris* Culture (% *v*/*v*)		FLX Solution (% *v*/*v*)	Medium OECD ^1^ (% *v*/*v*)
		Free Cells	Immobilized Cells		
Main experiment	72	20	0 (390 ± 1 beads)	8	0
1st control	72	20	0 (390 ± 1 beads)	0	8
2nd control	72	0	0	8	20
3rd control	0	0	0	8	92
4th control	0	20	0 (390 ± 1 beads)	0	80
5th control	72	0	0	0	28

^1^ OECD: Organization for Economic Co-operation and Development.

**Table 3:** Elemental analysis of living and non-living *C. vulgaris* biomass before and after FLX uptake.

*Chlorella vulgaris* Sample		Element Mass Fraction (%)									
		C	N	O	Na	Mg	Si	P	S	K	Ca
Living	Before FLX biosorption	55.1	----	34.3	0.8	0.7	3.3	2.1	1.2	1.6	1.0
	After FLX biosorption	47.8	8.4	39.0	----	0.5	0.6	1.0	0.8	1.2	0.7
Lyophilized	Before FLX biosorption	56.4	----	38.2	0.5	0.8	----	1.7	1.0	0.5	0.9
	After FLX biosorption	54.3	----	40.1	0.4	0.6	1.2	1.1	1.4	----	1.1

**Table 4:** Parameters of kinetic models for FLX biosorption onto the living and non-living *C. vulgaris* biomass.

Model	Parameters ^1^	Living *C. vulgaris* Biomass	Non-Living *C. vulgaris* Biomass
**Elovich**			
	*α* (mg·g^−1^·min^−1^)	4 ± 3	7 × 10^9^ ± 9 × 10^10^
	*β* (g·mg^−1^)	8 ± 1	12 ± 6
	SSE	0.262	0.440
	$S_{y.x}$	0.099	0.177
	$\;R_{adj}^{2}$	0.827	0.913
	$\chi_{red}^{2}$	0.0097	0.0314
	*AIC*	−130	−52
	*BIC*	−126	−49
**Pseudo-first-order**			
	$q_{e}$ (mg·g^−1^)	0.96 ± 0.02	2.33 ± 0.04
	*k*_1_ (min^−1^)	0.20 ± 0.02	0.7 ± 0.1
	SSE	0.217	0.317
	$S_{y.x}$	0.090	0.150
	$R_{adj}^{2}$	0.857	0.938
	$\chi_{red}^{2}$	0.0080	0.0226
	*AIC*	−136	−57
	*BIC*	−132	−54
**Pseudo-second order**		**Best-fit**	**Best-fit**
	$q_{e}$ (mg·g^−1^)	1.03 ± 0.03	2.39 ± 0.05
	*k*_2_ (g·mg^−1^·min^−1^)	0.3 ± 0.1	0.7 ± 0.2
	SSE	0.173	0.276
	$S_{y.x}$	0.080	0.140
	$R_{adj}^{2}$	0.886	0.946
	$\chi_{red}^{2}$	0.0064	0.0197
	*AIC*	−142	−59
	*BIC*	−138	−57

^1^ *α*: Elovich constant related to the initial biosorption rate; *β*: Elovich constant related to the desorption rate; SSE: Sum of squares error; $S_{y.x}$: Standard error of estimate; $R_{adj}^{2}$: Adjusted R-square correlation coefficient; $\chi_{red}^{2}$: Reduced chi-squared; *AIC*: Akaike information criterion; *BIC*: Bayesian information criterion; $q_{e}$: Equilibrium biosorption capacity; *k*_1_: Pseudo-first-order kinetic constant of the model; *k*_2_: Pseudo-second-order kinetic constant.

**Table 5:** Parameters of equilibrium models for FLX biosorption onto the living and non-living *C. vulgaris* biomass.

Model	Parameters ^1^	Living *C. vulgaris* Biomass	Non-Living *C. vulgaris* Biomass
**Freundlich**			
	$n_{F}$	2.7 ± 0.3	1.8 ± 0.2
	$K_{F}$ ((mg·g^−1^)·(L·mg^−1^)^1:*n*^*_F_*)	2.8 ± 0.3	2.8 ± 0.4
	SSE	0.228	0.066
	$S_{y.x}$	0.151	0.091
	$R_{adj}^{2}$	0.923	0.961
	$\chi_{red}^{2}$	0.0228	0.0083
	*AIC*	−42	−44
	*BIC*	−10	−43
**Langmuir**		**Best-fit**	**Best-fit**
	$q_{mL}$ (mg·g^−1^)	1.9 ± 0.1	1.6 ± 0.2
	$K_{L}$ (L·mg^−1^)	20 ± 5	11 ± 3
	SSE	0.175	0.043
	$S_{y.x}$	0.132	0.073
	$R_{adj}^{2}$	0.941	0.975
	$\chi_{red}^{2}$	0.0175	0.0054
	*AIC*	−45	−48
	*BIC*	−43	−48
**Langmuir**−**Freundlich**			
**(Sips)**	$q_{mLF}$ (mg·g^−1^)	2.1 ± 0.5	1.5 ± 0.4
	$n_{LF}$	0.8 ± 0.3	1.1 ± 0.3
	$K_{LF}$ (L·mg^−1^)	15 ± 10	13 ± 8
	SSE	0.170	0.043
	$S_{y.x}$	0.137	0.078
	$R_{adj}^{2}$	0.936	0.971
	$\chi_{red}^{2}$	0.0189	0.0061
	*AIC*	−43	−47
	*BIC*	−41	−45
**Redlich**−**Peterson**			
	$K_{RP}$ (L·g^−1^)	53 ± 30	20 ± 10
	$a_{RP}$ (L·mg^−1^)*^β^_RP_*	24 ± 11	11 ± 3
	$\beta_{RP}$	0.9 ± 0.2	0.9 ± 0.4
	SSE	0.162	0.043
	$S_{y.x}$	0.134	0.078
	$R_{adj}^{2}$	0.939	0.971
	$\chi_{red}^{2}$	0.0181	0.0061
	*AIC*	−44	−47
	*BIC*	−42	−45
**Tóth**			
	$q_{mT}$ (mg·g^−1^)	2 ± 1	2 ± 1
	$n_{T}$	0.7 ± 0.5	0.9 ± 0.7
	$K_{T}$ (L·mg^−1^)	0.1 ± 0.1	0.1 ± 0.2
	SSE	0.167	0.043
	$S_{y.x}$	0.136	0.078
	$R_{adj}^{2}$	0.938	0.971
	$\chi_{red}^{2}$	0.0185	0.0061
	*AIC*	−43	−47
	*BIC*	−41	−45

^1^*C_e_*: Equilibrium concentration; $q_{mL}$: Langmuir constant related to the maximum biosorption capacity considering monolayer coverage; $K_{L}$: Langmuir constant related to the energy of biosorption, respectively; $n_{F}$: Freundlich constant related to biosorption intensity; $K_{F}$: Freundlich constant related to biosorption capacity; $q_{mLF}$: Langmuir–Freundlich maximum biosorption capacity; $n_{LF}$ and $K_{LF}$: Langmuir–Freundlich constants; $K_{RP}$ and $a_{RP}$: Redlich–Peterson parameters; $\beta_{RP}$: Exponential value of 0–1; $q_{mT}$: Tóth maximum biosorption capacity; $n_{T}$ and $K_{T}$: Tóth constants.

**Table 6:** Parameters of equilibrium models for FLX biosorption onto living and non-living *C. vulgaris* biomass.

Sorbent	Experimental Conditions		$q_{m}$ (mg·g^−1^)	References
	Time (min)	Temperature (°C)		
Commercial activated carbon	<360	25	96.2	[61]
Papermill sludge-based non-activated carbon	<360	25	120.4	[61]
Papermill sludge-based activated carbon with ZnCl_2_	<360	25	28.4	[61]
Papermill sludge-based activated carbon with NaOH	<360	25	136.6	[61]
Eucalyptus biochar	15	~20	6.41	[62]
Hollow trees biochar	15	~20	3.04	[62]
Vine biochar	15	~20	2.80	[62]
Synthetic zeolite 13×	>600	25	32.11	[63]
Synthetic zeolite 4A	>1200	25	21.86	[63]
Spent coffee grounds	180–600	25	14.31	[63]
Pine bark	180–600	25	6.53	[63]
Cork waste	180–600	25	4.74	[63]
Non-living *Bifurcaria bifurcata* biomass	180	~20	6.81	[19]
Living *Chlorella vulgaris* biomass	120	~20	1.9	This study
Non-living *Chlorella vulgaris* biomass	120	~20	1.6	This study

$q_{m}$: Maximum monolayer adsorption capacity.

## Data Availability

Not applicable.

## Supplementary Materials

The following supporting information can be downloaded at: https://www.mdpi.com/article/10.3390/ijerph19106081/s1. Table S1: Physicochemical properties of fluoxetine (FLX) hydrochloride; Table S2: List of reagents used in the study; Table S3: List of equations; Table S4: Characteristics of the treated municipal wastewaters used to evaluate the simultaneous removal of FLX and nutrients by living *Chlorella vulgaris*, free and immobilized; Table S5: Band assignments of FT-IR spectra of living and non-living *Chlorella vulgaris* biomass before and after FLX uptake; Figure S1: Energy dispersive spectroscopy (EDS) graphs of living C. vulgaris biomass (a1) before and (a2) after FLX uptake, and non-living *Chlorella vulgaris* biomass (b1) before and (b2) after FLX uptake (secondary electrons (SE); ×3000; 15 kV; working distance (WD) = 10.7 mm); Figure S2: Species distribution diagram of FLX as a function of pH (adapted from [51]); Figure S3: pH value at the beginning (day 0) and the end (day 9) of the main experiment and control assays, for free *Chlorella vulgaris.* Different letters indicate significant differences among assays; Figure S4: pH value at the beginning (day 0) and the end (day 9) of the main experiment and control assays, for immobilized *Chlorella vulgaris*. Different letters indicate significant differences among assays. References [29,30,37,38,39,40,41,42,43,44,45,46,47,51] are cited in the supplementary materials.
