# Supplementary material for: Fluoxetine and Nutrients Removal from Aqueous Solutions by Phycoremediation
Source: Int J Environ Res Public Health. 2022 May 17;19(10):6081. doi: 10.3390/ijerph19106081 (PMC9141300; doi:10.3390/ijerph19106081)
Supplement: Supplementary file 1 [file ijerph-19-06081-s001.zip › ijerph-1692227-supplementary.pdf]

International Journal of
Environmental Research and Public Health

MDPI

SUPPLEMENTARY MATERIALS

Fluoxetine and Nutrients Removal from Aqueous Solutions by Phycoremediation

Andreia D. M. Silva, Diana F. Fernandes, Sónia A. Figueiredo *, Olga M. Freitas * and Cristina Delerue-Matos

REQUIMTE/LAQV- Associated Laboratory for Green Chemistry (LAQV) of the Network of Chemistry and Technology (REQUIMTE), Instituto Superior de Engenharia do Porto - Politécnico do Porto, Rua Dr. António Bernardino de Almeida 431, 4249-015 Porto, Portugal; andreia.silva@graq.isep.ipp.pt (A.D.M.S.); 1140207@isep.ipp.pt (D.F.F.); cmm@isep.ipp.pt (C.D.-M.)

* Correspondence: saf@isep.ipp.pt (S.A.F.); omf@isep.ipp.pt (O.M.F.)

Contents

Page 2 –

Table S1. Physicochemical properties of fluoxetine (FLX) hydrochloride.

Table S2. List of reagents used in the study.

Page 3 –

Table S3. List of equations.

Page 4 –

Table S4. Characteristics of the treated municipal wastewaters used to evaluate the simultaneous removal of FLX and nutrients by living *Chlorella vulgaris*, free and immobilized.

Table S5. Band assignments of FT-IR spectra of living and non-living *Chlorella vulgaris* biomass before and after FLX uptake.

Page 5 –

Figure S1. . Energy Dispersive Spectroscopy (EDS) graphs of living C. vulgaris biomass (a1) before and (a2) after FLX uptake, and non-living Chlorella vulgaris biomass (b1) before and (b2) after FLX uptake (Secondary Electrons (SE); x3000; 15 kV; Working Distance (WD)=10.7 mm).

Figure S2. Species distribution diagram of FLX as a function of pH (adapted from [1]).

Page 6 –

Figure S3. pH value at the beginning (day 0) and the end (day 9) of the main experiment and control assays, for free *Chlorella vulgaris*. Different letters indicate significant differences among assays.

Figure S4. pH value at the beginning (day 0) and the end (day 9) of the main experiment and control assays, for imobilized *Chlorella vulgaris*. Different letters indicate significant differences among assays.

References

Table S1. Physicochemical properties of fluoxetine (FLX) hydrochloride.

Molecular formula [(1)]	Molecular structure	M.w. ($g\,mol^{-1}$)	S.w. ($mg\,L^{-1}$)	*p*Ka [(1)]	Log K_{ow} [(1)]	*H* [(2)] ($Pa\,m^3\,mol^{-1}$)
$C_{17}H_{18}F_3NO \cdot HCl$	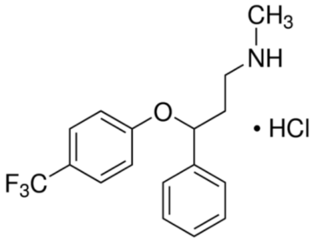	345.79	4000	9.8	4.17	2.46×10^{-5}

M.w.: Molecular weight; S.w.: Solubility in water; Ka: Acid dissociation constant; K_{ow}: Octanol-water partition coefficient; *H*: Henry's law constant.

[(1)] Source [2]; [(2)] Source [3].

Table S2. List of reagents used in the study.

Reagent	Supplier	Country
Potassium bromide (KBr, for IR spectroscopy)	PanReac	Spain
Sodium chloride (NaCl, purity 99.9%)	VWR International™	United Kingdom
Sodium hydroxide (NaOH, purity >99.0%)	Labkem	Spain
Hydrochloric acid (HCl, purity 37% wt)	Honeywell Fluka™	Austria
Alginic acid sodium salt from brown algae (algin, sodium alginate)	Sigma-Aldrich	United Kingdom
Calcium chloride dihydrate ($CaCl_2 \cdot 2\,H_2O$, purity >99.0%)	Merck	Germany
Tri-sodium citrate dihydrate ($C_6H_5Na_3O_7 \cdot 2\,H_2O$, purity >99.0%)	Merck	Germany
Acetonitrile (CH_3CN, purity 99.99%)	VWR BDH® Chemicals HPLC-super gradient	France
Formic acid (HCOOH, purity 99%)	CARLO ERBA Reagents	France
Methanol (CH_3OH, purity >99.9%)	VWR BDH® Chemicals HPLC-super gradient	Netherlands
Molybdate reagent	HACH	USA
Amino acid reagent	HACH	USA
Potassium persulfate ($K_2S_2O_8$, purity 99%)	Merck	Germany

Table S3. List of equations.

Equation	Numbering	References
$DW = \left(8.27 \times 10^{-5} \cdot FI_{485/645} \pm 0.41 \times 10^{-5}\right) + (-7.14 \times 10^{-1} \pm 0.70 \times 10^{-1}), \quad R^2 = 0.993$	S1	N.A.
$DW = \left(2.553 \times 10^{-5} \cdot FI_{485/645} \pm 0.099 \times 10^{-5}\right) + (6.6 \times 10^{-3} \pm 3.0 \times 10^{-3}), \quad R^2 = 0.991$	S2	N.A.
$Peak\ Area = (329.3 \cdot [FLX] \pm 0.6) + (861 \pm 371), \quad R^2 = 0.99996$	S3	N.A.
$q_t = (C_0 - C_t) \cdot \frac{V}{m},$	S4	N.A.
$\text{Removal (\%)} = \frac{C_0 - C_t}{C_0} \cdot 100,$	S5	N.A.
$q_t = \frac{1}{\beta} \cdot \ln(1 + \alpha \cdot \beta \cdot t),$	S6	[4]
$q_t = q_e \cdot (1 - e^{-k_1 \cdot t}),$	S7	[5]
$q_t = \frac{q_e^2 \cdot k_2 \cdot t}{1 + q_e \cdot k_2 . t},$	S8	[6]
$q_e = K_F \cdot {C_e}^{1/n_F},$	S9	[7]
$q_e = \frac{q_{mL} \cdot K_L \cdot C_e}{1 + K_L \cdot C_e},$	S10	[8]
$q_e = \frac{q_{mLF} \cdot (K_{LF} . C_e)^{n_{LF}}}{1 + (K_{LF} \cdot C_e)^{n_{LF}}},$	S11	[9]
$q_e = \frac{K_{RP} \cdot C_e}{1 + a_{RP} \cdot C_e^{\beta_{RP}}},$	S12	[10]
$q_e = \frac{q_{mT} \cdot K_T \cdot C_e}{[1 + (K_T \cdot C_e)^{n_T}]^{1/n_T}},$	S13	[11]
$\text{Inhibition Rate (\%)} = \frac{Growth_{4th\ control} - Growth_{Main\ experiment}}{Growth_{4th\ control}} \cdot 100$	S14	N.A.

DW: dry weight; $FI_{485/645}$: fluorescence intensity of chlorophyll; $[FLX]$: fluoxetine concentration; q_t: biosorption capacity; C_0: initial FLX concentration; C_t: FLX concentration at a given time; V: volume of the solution t; m: mass of the *Chlorella vulgaris* biomass; β: Elovich constant related to the desorption rate; t: time; α: Elovich constant related to the initial biosorption rate; q_e: equilibrium biosorption capacity; k_1: pseudo-first-order kinetic constant of the model; k_2: pseudo-second-order kinetic constant; h_0: initial biosorption rate; C_e: equilibrium concentration; q_{mL}: Langmuir constant related to the maximum biosorption capacity considering monolayer coverage; K_L: Langmuir constants related to the energy of biosorption, respectively; n_F: Freundlich constant related to biosorption intensity; K_F: Freundlich constant related to biosorption capacity; q_{mLF}: Langmuir-Freundlich maximum biosorption capacity; n_{LF} and K_{LF}: Langmuir-Freundlich's constants; K_{RP} and a_{RP}: Redlich-Peterson's parameters; β_{RP}: exponential value that goes 0-1; q_{mT}: Tóth maximum biosorption capacity; n_T and K_T: Tóth constants; N.A.: not aplicable.

Table S4. Characteristics of the treated municipal wastewaters used to evaluate the simultaneous removal of FLX and nutrients by living *Chlorella vulgaris*, free and immobilized.

Wastewater parameters	Free *Chlorella vulgaris*	Immobilized *Chlorella vulgaris*
**Biochemical oxygen demand (mg L^{-1} O_2)**	7	22
**Chemical oxygen demand (mg L^{-1} O_2)**	49	98
**Conductivity (μS cm^{-1})**	627	1402
**Total nitrogen (mg L^{-1})**	24.5	81.0
**Total phosphorus (mg L^{-1})**	2.2	25.5
**pH**	7.75	7.68

Table S5. Band assignments of FT-IR spectra of living and non-living *Chlorella vulgaris* biomass before and after FLX uptake.

Band	Wavenumber (cm^{-1})	Band assignments [1]	References
A	~3438	ν(O-H) (associated) to water molecules or hydroxyl radicals of polysaccharides<br>ν(N-H) (associated) to proteins (amide A)	[12, 13]
B<br>C	~2923<br>~2855	$\nu_{as}(CH_2)$ and $\nu_s(CH_2)$ of lipids and carbohydrates	[12, 13]
D	~1739	ν(C=O) of esters associated with fatty acids and cellulose	[12, 14]
E	~1647	ν(C=O) of amides I band associated with proteins	[12, 13]
F	~1565	δ(N-H) of amides II band and δ(C-N) of proteins	[12, 13]
G	~1462	$\delta_{as}(CH_2)$ of lipids<br>$\delta_{as}(CH_2)$ and $\delta_{as}(CH_3)$ of proteins	[12, 14]
H	~1417	$\delta_s(N(CH_3)_3)$ of lipids<br>$\delta_s(CH_2)$ and $\delta_s(CH_3)$ of proteins<br>ν_s(C-O) of COO^- of carboxylic groups	[12, 14]
I	~1387	$\delta_s(N(CH_3)_3)$ of lipids<br>$\delta_s(CH_2)$ and $\delta_s(CH_3)$ of proteins<br>ν_s(C-O) of COO^- of carboxylic groups	[12, 14]
J	~1250	ν_{as}(>P=O) of phosphodiester backbone from nucleic acids and phospholipids	[12, 14]
K<br>L	~1080<br>~1049	ν_{as}(>P=O) of phosphodiester backbone from nucleic acids<br>ν(C-O) and ν(C-C) and of carbohydrates<br>δ(C-O-H) and δ(C-O-C) of carbohydrates	[12, 13]

[1] ν: stretching; ν_s: symmetric stretching; ν_{as}: asymmetric stretching; δ: deformation; δ_s: symmetric deformation; δ_{as}: asymmetric deformation.

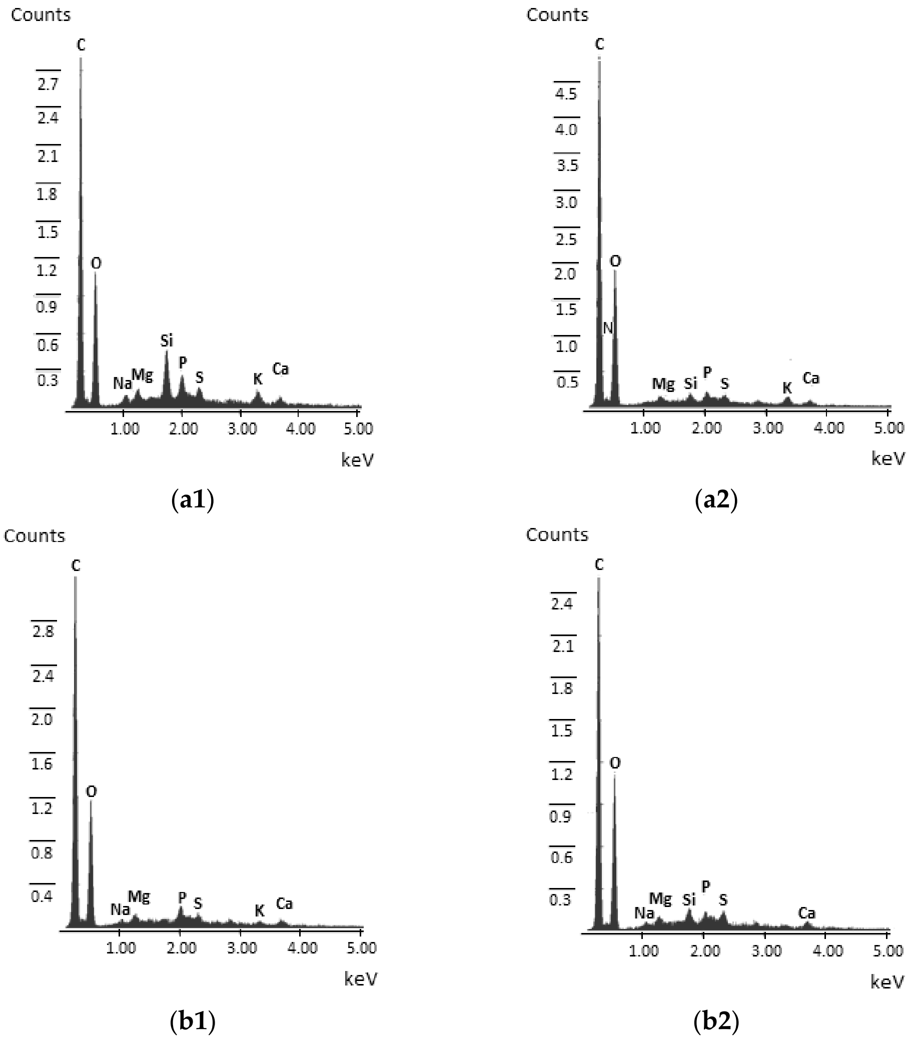

Figure S1. Energy Dispersive Spectroscopy (EDS) graphs of living *C. vulgaris* biomass (a1) before and (a2) after FLX uptake, and non-living *Chlorella vulgaris* biomass (b1) before and (b2) after FLX uptake (Secondary Electrons (SE); x3000; 15 kV; Working Distance (WD)=10.7 mm).

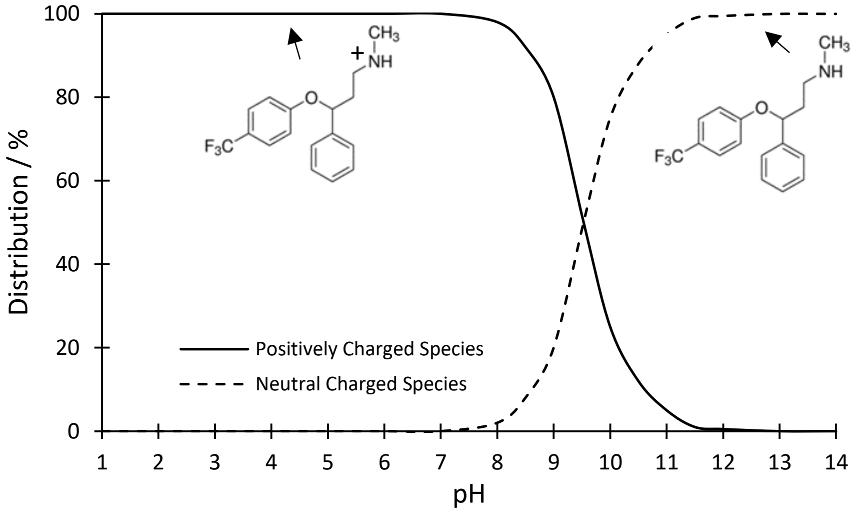

Figure S2. Species distribution diagram of FLX as a function of pH (adapted from [1]).

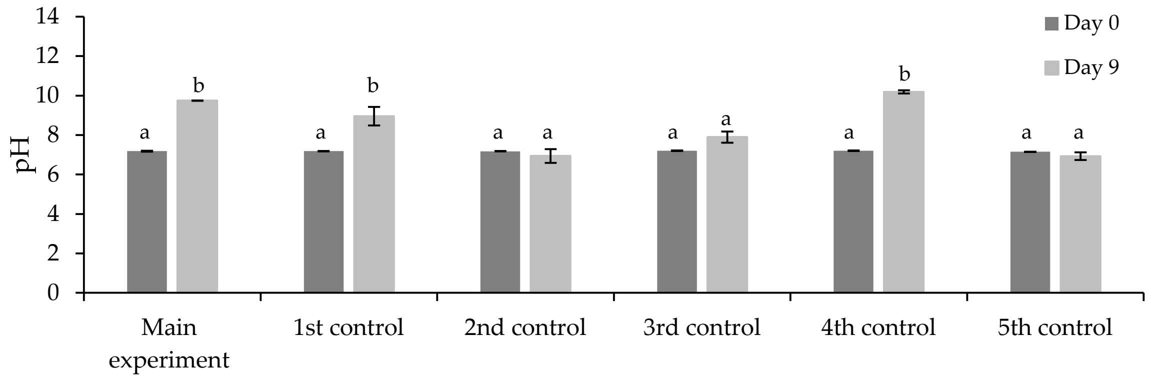

Figure S3. pH value at the beginning (day 0) and the end (day 9) of the main experiment and control assays, for free *Chlorella vulgaris*. Different letters indicate significant differences among assays.

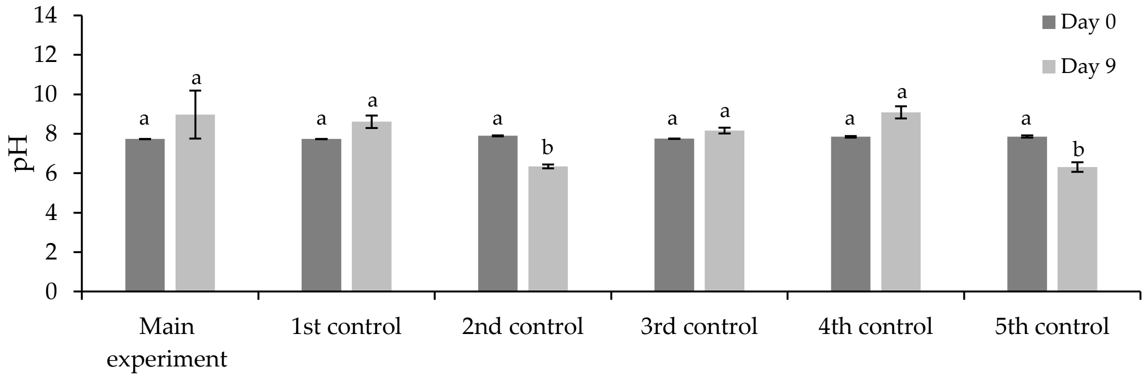

Figure S4. pH value at the beginning (day 0) and the end (day 9) of the main experiment and control assays, for immobilized *Chlorella vulgaris*. Different letters indicate significant differences among assays.

References

1. Schneider, J.D. pH Effects on the Sorption of Fluoxetine and Sulfamethoxazole by Three Powdered Activated Carbons, in Civil and Environmental Engineering. Master's Thesis, Utah State University, Logan, UT, USA, May 2018.
2. ChemAxon. Marvin Software. Available online: https://chemaxon.com (accessed on 10 January 2019).
3. AMAP Chemicals. Fluoxetine. Available online: https://chemicals.amap.no/chemicals/fluoxetine (accessed on 10 January 2019).
4. Low, M.J.D. Kinetics of Chemisorption of Gases on Solids. *Chem. Rev.* **1960**, *60*, 267–312. https://doi.org/10.1021/cr60205a003.
5. Lagergren, S. About theory of so-called adsorption of soluble substances. *K. Sven. Vetensk. Handl.* **1898**, *24*, 1–39.
6. Ho, Y.S.; McKay, G. Pseudo-second order model for sorption processes. *Process Biochem.* **1999**, *34*, 451–465. https://doi.org/10.1016/S0032-9592(98)00112-5.
7. Freundlich, H.M.F. Über die Adsorption in Lösungen. *Z. Für Elktrochemie Angew. Phys. Chemie* **1906**, *57*, 385–470. https://doi.org/10.1515/zpch-1907-5723.
8. Langmuir, I. The Adsorption of Gases on Plane Surfaces of Glass, Mica and Platinum. *J. Am. Chem. Soc.* **1918**, *40*, 1361–1403. https://doi.org/10.1021/ja02242a004.
9. Sips, R. On the Structure of a Catalyst Surface. *J. Chem. Phys.* **1948**, *16*, 490–495. https://doi.org/10.1063/1.1746922.

10. Redlich, O.; Peterson, D.L. A Useful Adsorption Isotherm. *J. Phys. Chem.* **1959**, *63*, 1024–1024. https://doi.org/10.1021/j150576a611.
11. Tóth, J. State Equation of the Solid-gas Interface Layers. *Acta Chim. Acad. Sci. Hung.* **1971**, *69*, 311–328.
12. Duygu. D.Y.; Udoh, A.U.; Ozer, T.B.; Akbulut, A.; Erkaya, I.A.; Yildiz, K.; Guler, D. Fourier transform infrared (FTIR) spectroscopy for identification of *Chlorella vulgaris* Beijerinck 1890 and *Scenedesmus obliquus* (Turpin) Kützing 1833. *Afr. J. Biotechnol.* **2012**, *11*, 3817–3824. https://doi.org/10.5897/AJB11.1863.
13. Ponnuswamy, I.; Soundararajan, M.; Syed, S. Isolation and Characterization of Green Microalgae for Carbon Sequestration, Waste Water Treatment and Bio-fuel Production. *Int. J. Bio-Sci. Bio-Technol.* **2013**, *5*, 17–26.
14. Grace, C.E.E.; Lakshmi, P.K.; Meenakshi, S.; Vaidyanathan, S.; Srisudha, S.; Mary, M.B. Biomolecular transitions and lipid accumulation in green microalgae monitored by FTIR and Raman analysis. *Spectrochim. Acta A Mol. Biomol. Spectrosc* **2020**, *224*, 117382. https://doi.org/10.1016/j.saa.2019.117382.

.

.
